# Epidemiological trends and age–period–cohort analysis of alcoholic liver cancer from 1990 to 2021

**DOI:** 10.1007/s12072-025-10966-5

**Published:** 2025-12-20

**Authors:** Jiajia Chen, Runhua Li, Xiaojie Li, Kai Shi, Rui Zeng, Kunpeng Hu, Qingliang Wang

**Affiliations:** 1 Department of Hepatobiliary Surgery, Chaozhou Central Hospital, Chaozhou, 521000 China; 2 Department of Cancer Prevention, Zhejiang Cancer Hospital, Hangzhou Institute of Medicine (HIM), Chinese Academy of Sciences, Hangzhou, 310022 China; 3 Department of Laboratory Medicine, The Third Affiliated Hospital, Sun Yat-Sen University, Guangzhou, 510630 China; 4 Department of Hepatobiliary-Pancreatic-Splenic Surgery, The Third Affiliated Hospital, Sun Yat-Sen University, Tianhe Road 600, Guangzhou, 510630 China; 5 Department of Thyroid and Breast Surgery, The Third Affiliated Hospital, Sun Yat-Sen University, Tianhe Road 600, Guangzhou, 510630 China

**Keywords:** Liver cancer, Global burden of disease, Alcohol, Incidence, Mortality, Epidemiological changes, Age-standardized rate, Decomposition analysis, Age–period–cohort analysis, Joinpoint regression

## Abstract

**Background:**

The disease burden of alcoholic liver cancer (ALC) has been changing due to socioeconomic development. An up-to-date evaluation of this change can increase public awareness and facilitate health policy development.

**Methods:**

The data were extracted from the Global Burden of Diseases Study (GBD) 2021. Epidemiological changes of morbidity and mortality in different regions, as well as the differences in gender and age were analyzed. Temporal trends were determined using Joinpoint regression analysis and age–period–cohort analysis was employed to evaluate the effects of age, period, and cohort.

**Results:**

Globally, there were 99,543.67 new cases (95% UI 80957.40–120401.87) and 92,227.78 deaths (95% UI 75053.11–112,160.27) in 2021, increased by 158.92% and 141.61%, respectively, compared to 1990. Decomposition analysis revealed that population growth was the primary driver of the increased morbidity (41.13%) and mortality (54.87%). The global estimated annual percentage change (EAPC) of morbidity and mortality exhibited an ascending trend, while differences existed across different regions. The morbidity and mortality rates were higher in males than in females across all age groups. Joinpoint regression analysis showed an increased morbidity and mortality rate. The age effect indicated that the highest risk was observed at 75–79 years for morbidity and 80–84 years for mortality. The period effect showed an increasing risk, while improving cohort risks for mortality were identified.

**Conclusion:**

Alcoholic liver cancer has posed an increasing health burden since 1990, particularly affecting male and the elderly population. Effective prevention policies on specific age groups should be formulated to improve this situation.

**Graphical abstract:**

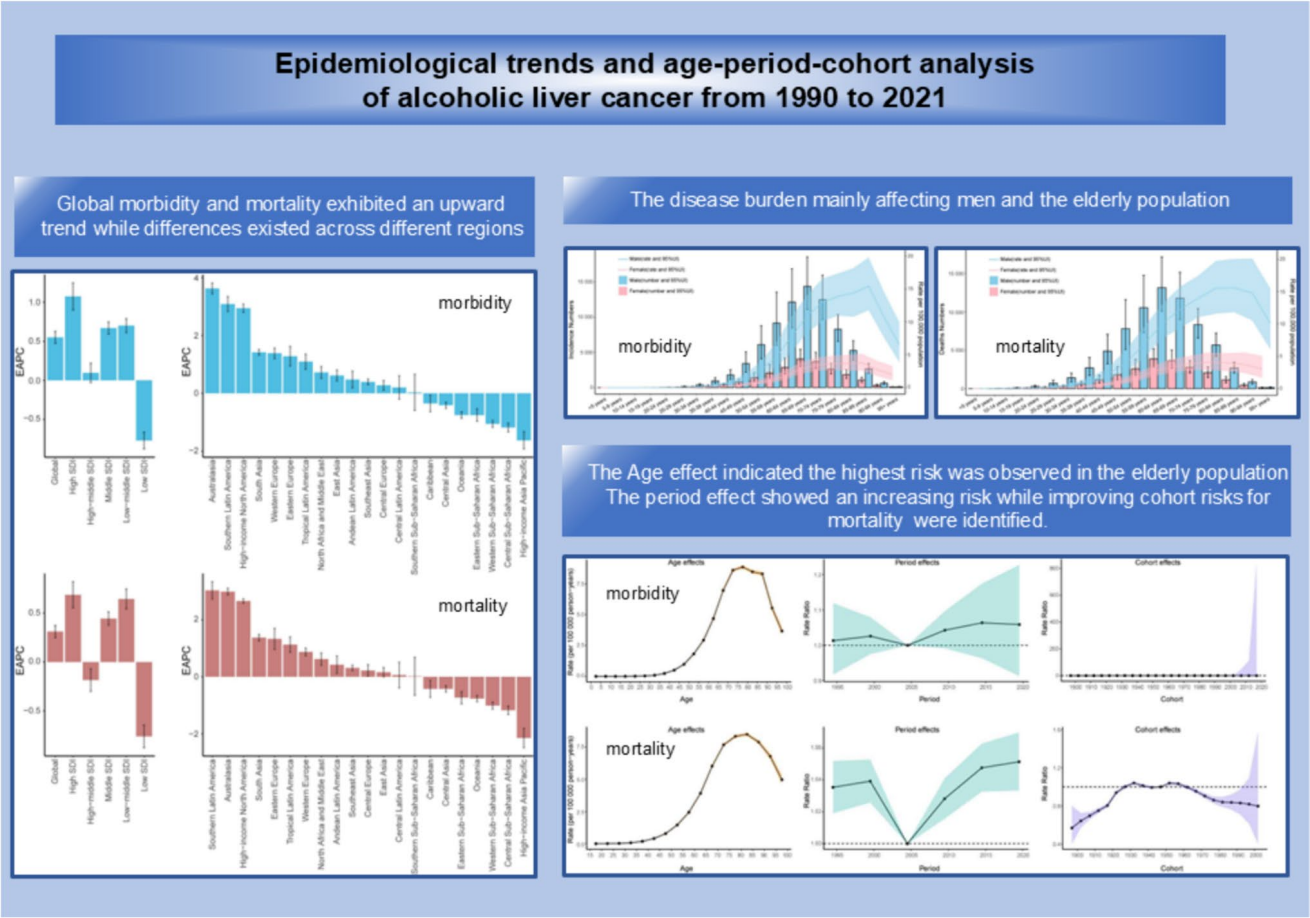

**Supplementary Information:**

The online version contains supplementary material available at 10.1007/s12072-025-10966-5.

## Introduction

Liver cancer remains a formidable global health challenge, representing a leading cause of cancer-related morbidity and mortality worldwide [1]. Approximately, 529,202 newly diagnosed liver cancer cases and 483,875 deaths globally were recorded in 2021 with an age-standardized prevalence rate 8.68 [2, 3]. Liver cancer is usually the result of multiple factors in the context of chronic liver disease. Alcohol consumption stands as another major, modifiable risk factor for liver cancer by acting independently or synergistically with other risk factors. Excessive alcohol consumption has been reported as the second most independent risk factor for liver cancer after viral infection [4].

Global economic development and concomitant lifestyle changes are continuously reshaping patterns of alcohol consumption across populations [5]. Besides, national policies and marketing strategies have influenced both the prevalence of drinking and the levels of alcohol intake in many regions [6]. These evolving patterns suggest that the liver diseases due to alcohol use are not static but subject to significant temporal and geographical variation. It has been reported that alcohol consumption varies by age and location [7], and understanding these dynamics is crucial for anticipating future trends and targeting interventions effectively.

The economic impact of alcoholic liver diseases is substantial, and it will incur an estimated cost of $880 billion between 2022 and 2040 [8]. Besides, alcoholic liver cancer is associated with lower surveillance rate, more advanced stage, and poorer survival outcomes [9]. It has direct costs to the health care system in terms of medical treatment and indirect costs to social development attributable to the loss of labor productivity. Thus, quantifying the precise contribution of alcohol to total liver cancer, delineating how this burden has evolved are essential steps for public health planning and resource allocation.

Based on recent study, ALC poses a significant global burden, particularly in economically advanced regions with males and middle-aged adults as high-risk groups [10]. The prevalence of primary liver cancer from alcohol increased with annual percent change of 2.93% among older adults in the United Sates from 2000 to 2021 [11]. Despite these advancements, an updated assessment of epidemiological trends is needed, especially to explore the key drivers of changing trends. In this current study, we comprehensively analyzed the alcoholic liver cancer burden. By assessing the epidemiological trends and performing age–period–cohort analysis, we aimed to increase public awareness of the evolving global challenge of alcoholic liver cancer, and update prevention strategies to control these diseases.

## Methods

### Data source and disease definition

The burden data were downloaded from the GBD study 2021. Based on the SDI value, the 204 countries and territories were divided into five categories (low, low–middle, middle, high–middle, and high SDI). It was also stratified into 21 regions based on the GBD region classification. According to the International Classification of Diseases tenth edition (ICD-10), alcohol-related liver disease was classified using codes K70-K70.3, while liver cancer corresponded to ICD-10 code C22-C22.4, C22.7-C22.8. To simplify disease categorization, the “ICD code groups” were applied by the GBD Study 2021 and mapped to a disease or injury modeling entity by GBD modelers. DisMod-MR 2.1 (Disease Modelling Meta-Regression; version 2.1) was used to distinguish the proportions of liver cancer due to underlying etiologies. After data identification and adjustments, the inclusion criteria for ALC were strictly applied, while other etiologies were excluded by researchers in GBD study 2021.

### Estimation of the burden of alcoholic liver cancer

The proportion of ALC in different etiology types was explored. The number and age-standardized rate of incidence, prevalence, DALYs, and deaths were acquired online and presented with 95% uncertainty intervals (UI). The estimated annual percentage change (EAPC) across different regions was also analyzed to measure the alcoholic liver cancer burden. The morbidity and mortality burden stratified by gender and age were also explored. The population was divided into 20 age groups (every 5 years according to age) to summarize the age distribution of the burden.

### Decomposition analysis

Decomposition analysis was employed to assess the global morbidity and mortality changes between 1990 and 2021. Decomposition analysis is a valuable tool for disentangling complex factors that contribute to observed changes over time. Based on the Das Gupta methodology, the contribution of each factor to the overall changes was quantified [12]. A robust decomposition method was used in our study. Details about the decomposition method have been described in the previous studies [13, 14]. The change can be attributed to population aging (A), population growth (P), and the changes in age-specific rates (M, epidemiological changes) using the following formula:

$${\mathrm{A}}\;{ = }\;{\mathrm{Ma}}\;{ + }\;{\mathrm{Iam}}\;{ + }\;{\mathrm{Ipa}}\;{ + }\;{\mathrm{Ipam}}$$

$${\mathrm{P}}\;{ = }\;{\mathrm{Mp}}\;{ + }\;{\mathrm{Ipm}}\;{ + }\;{\mathrm{Ipa}}\;{ + }\;{\mathrm{Ipam}}$$

$${\mathrm{M}}\;{ = }\;{\mathrm{Mm}}\;{ + }\;{\mathrm{Ipm}}\;{ + }\;{\mathrm{Iam}}\;{ + }\;{\mathrm{Ipam}}{.}$$

The Ma, Mp, and Mm indicate the main effects of population aging, population growth, and the change in morbidity or mortality rate, respectively; Ipa, Ipm, Iam, and Ipam denote the 2-way and 3-way interactions of the 3 components. The underlying drivers of change were gained after analysis. The disparity at the regional level among different SDI areas and 21 GBD regions was also compared.

### Temporal trend analysis

Joinpoint regression analysis was employed to examine the temporal trends of age-standardized incidence and death rates. Different trend changes were identified by segmenting the long-term trend into linear pieces, each representing a distinct phase of change. The analysis employs segmented regression on a log-linear regression model, and the optimal number of join points was determined using the Monte Carlo permutation test [15]. The annual percent change (APC) was calculated and presented with 95% confidence intervals (CIs) to assess trends. Trends were described as increasing or decreasing when the APC was statistically significant according to a two-sided *p* value < 0.05. The increasing trend was defined as the APC and its 95% CI are both > 0, while a decreasing trend is defined as APC and its 95% CI are both < 0. The whole analysis was carried out using the “Joinpoint” R package.

### Age–period–cohort analysis

The impact of age, period, and cohort effects on ALC was assessed by the age–period–cohort (APC) model, which was a widely accepted model in the field of epidemiological research. The methodological details were described in the previous literature [16]. We utilized 20 consecutive 5-year age intervals from 0 to 4 (< 5 years) to 95 + years for morbidity analysis and 17 consecutive 5-year age intervals ranging from 15–19 to 95 + years for mortality analysis. Six periods were divided from 1992 to 1996 (median 1994) to 2017–2021 (median 2019), with 2002–2006 as the reference period. For cohort effects, 25 consecutive birth cohorts were arranged, spanning from 1895 to 1899 (the 1897 cohort) to 2015–2019 (the 2017 cohort), with the 1955–1959 (the 1957 cohort) birth cohort serving as the incidence reference. Except for the birth cohorts 2007, 2012, and 2017, 22 consecutive birth cohorts were arranged for death analysis with the 1945–1949 (the 1947 cohort) birth cohort serving as the reference. The estimated parameters, longitudinal age curves, period relative risk, cohort relative risk, net drift, and local drift were calculated using the NIH APC Web tool [16]. The Wald chi-square test was adopted to assess the significance of estimable parameters and functions.

### Statistical analysis

The age-standardized rates were calculated using the direct method of standardization to the world population. All data analyses were conducted using the open-source software R (version 4.2.3) and JD_GBDR (Jing ding Medical Technology Co., Ltd.). A two-tailed *p* value < 0.05 was considered statistically significant.

## Results

### Changes in the proportion of ALC between 1990 and 2021

**Fig. 1:**
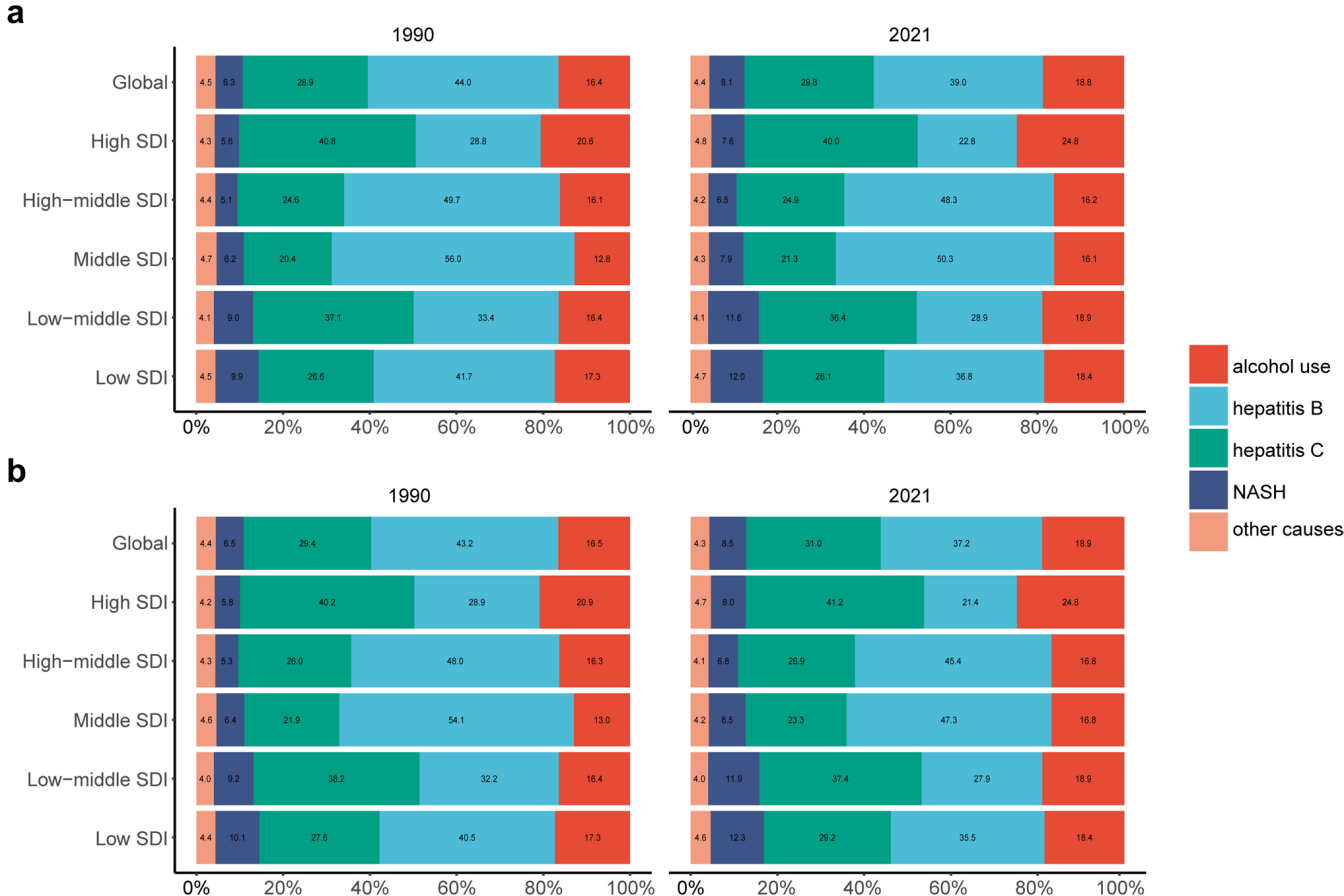
The contribution of alcohol use, hepatitis B, hepatitis C, NASH, and other causes of liver cancer in 1990 and 2021. The proportion of age-standardized incidence rate (**a**) and mortality rate (**b**) in global and different SDI regions. NASH nonalcoholic steatohepatitis, SDI Socio-demographic Index

Although with a decreasing trend, hepatitis B remains the largest component of the five etiologies in 2021, accounting for 39%, with an age-standardized incidence rate of 2.37 (95% UI 1.95–2.90). The age-standardized incidence rate of ALC was 1.14 (95% UI 0.93–1.37) in 2021, accounting for 18.8% of all cases. Compared to 1990, the percentage change of the proportion increased by 14.6%. In addition, the proportion of ALC presented an increasing tendency in all SDI regions, while the highest increase (25.8%) occurred in the middle SDI regions (Fig. 1a). The mortality presented a similar trend, alcoholic liver cancer accounts for 18.9%, with the age-standardized mortality rate 1.06 (95% UI 0.86–1.29) in 2021. The global percentage change increased by 14.5% compared to 1990, and the middle SDI regions had the highest growth rate, reaching 29.2% (Fig. 1b).

### The global burden of ALC in 1990 and 2021

Globally, the disease burden has increased significantly from 1990 to 2021. Specifically, the number of new cases rose from 38,445.23 in 1990 to 99,543.67 in 2021, while the number of deaths increased from 38,171.63 to 92,227.78. The age-standardized incidence and mortality rates presented an upward trend, with an EAPC of 0.55 and 0.31. The similar trends were also observed in prevalence and DALYs, and the numbers increased 194.40% and 122.24%, while the age-standardized rate increased 1.10 and 0.12, respectively (Table S1).

### Decomposition analysis

**Fig. 2:**
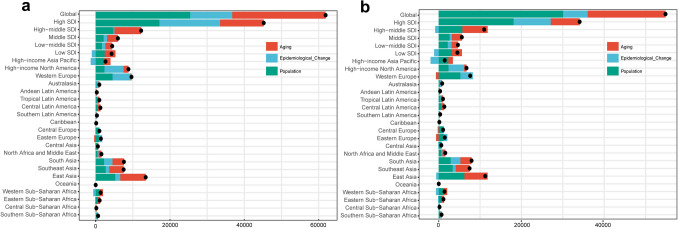
Decomposition analysis of incidence **a** and deaths and **b** burden of alcoholic liver cancer between 1990 and 2021 at the global and regional level. Black dots represent the total change contributed by aging, population growth, and epidemiological changes

Globally, the increased incidence was primarily attributable to population growth (41.13%) followed by population aging (40.61%). Among the 21 GBD regions, for the increased burden of morbidity, the highest contribution of aging was in Central Sub-Saharan Africa (87.15%), the highest contribution of population growth was in High-income Asia Pacific (86.55%), and the highest contribution of epidemiologic changes was in Eastern Europe (62.05%) (Fig. 2a). For the global changes of death burden, the results revealed that the population growth was the main factor, accounting for 54.87%. It was also the largest contributor in low-SDI regions (70.13%) and high-SDI regions (53.16%). At the regional level, high-income Asia Pacific had the highest contributions to aging and population growth. Epidemiological change mainly exerted a negative impact on the deaths burden in eight GBD regions with the most obvious negative contribution in High-income Asia Pacific (−135.02%) (Fig. 2b).

### Regional differences in temporal trends

**Fig. 3:**
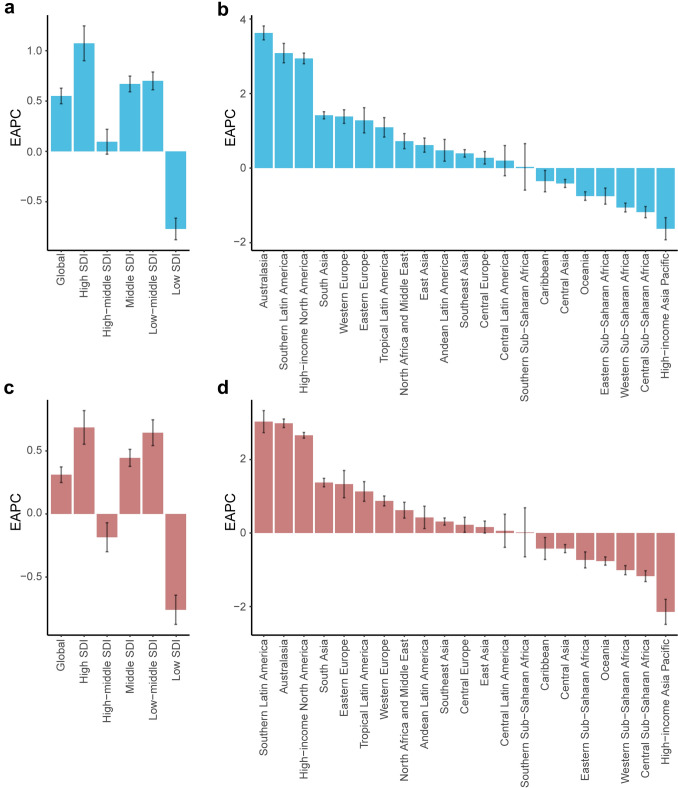
The estimated average percentage change (EAPC) of alcoholic liver cancer from 1990 to 2021. The EAPC of age-standardized incidence rate in global, different sociodemographic index regions (**a**) and the 21 GBD regions (**b**). The EAPC of age-standardized mortality rate in global, different sociodemographic index regions (c) and the 21 GBD regions (d)

Globally, the age-standardized morbidity and mortality both exhibited an ascending trend with EAPC value of 0.55 (95% CI: 0.47, 0.63) and 0.31(95% CI: 0.25, 0.37), respectively. An upward trend was identified across all SDI regions except for low-SDI regions. Among the 21 GBD regions, 14 GBD regions (66.7%) showed an increase in EAPC values, while 7 GBD regions had a decreased value. The most obvious increase occurred in Australasia with an EAPC value of 3.63, while the High-income Asia Pacific experienced the largest decline (EAPC = −1.63) (Fig. 3a). The high-SDI region also experienced the most increased mortality, with an EAPC value of 0.69, followed by the low–middle SDI region (0.64). For mortality, the largest increase was observed in Southern Latin America (EAPC = 3.03), and the largest decrease in the High-income Asia Pacific (EAPC = −2.15) (Fig. 3b).

### Age and gender differences in the disease burden of ALC

**Fig. 4:**
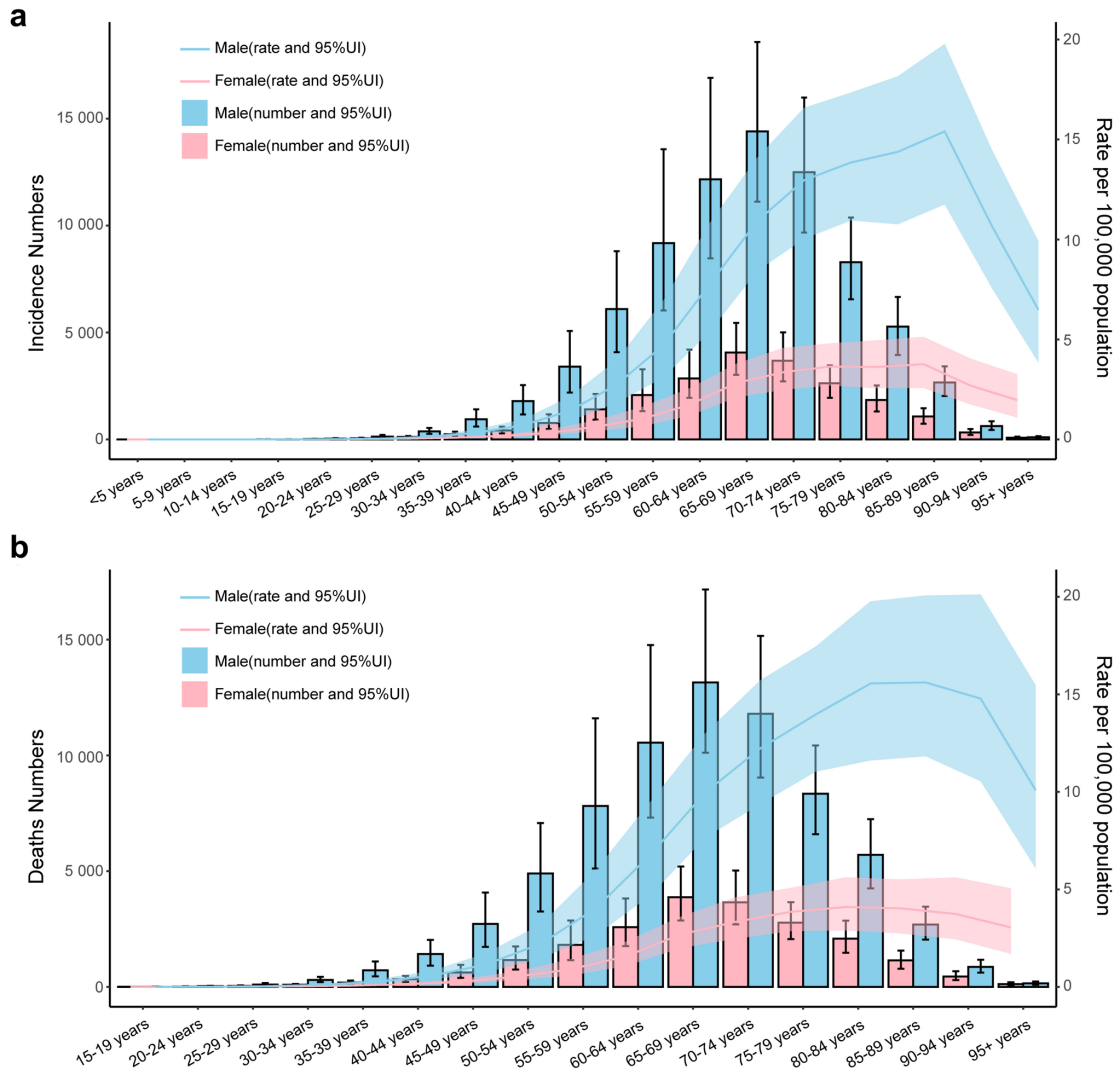
The incidence and mortality burden of alcoholic liver cancer in 2021. **a** The incidence cases and age-standardized incidence rate stratified by gender and different age groups; **b** the number of deaths and age-standardized mortality rate stratified by gender and different age groups

The age-standardized incidence rate for males was 1.14 in 2021, significantly higher than that for females (0.47) (Table S1). Among all age groups, the number of male cases was higher than that in female. The number of cases continues to increase with age, reaching a peak in the 65–69-year-old group. The age-standardized incidence rate also presented a similar trend, with a delayed peak to the 85–89-year-old group. The age-standardized incidence rate for males was 15.41 and for females was 3.76 (Fig. 4a). Further analysis was conducted to derive the gender and age differences in mortality burden. In 2021, the global age-standardized mortality rate for males (1.77) was also higher than that for females (0.45). The number of deaths was highest in the 65–69-year-old group, with 13,150.98 males and 3874.73 females. As for the age-standardized mortality rate, the highest burden was in the 85–89-year-old group for males (15.61) with an early peak in females (4.10) in the 80–84-year-old group (Fig. 4b).

### Temporal trends

**Fig. 5:**
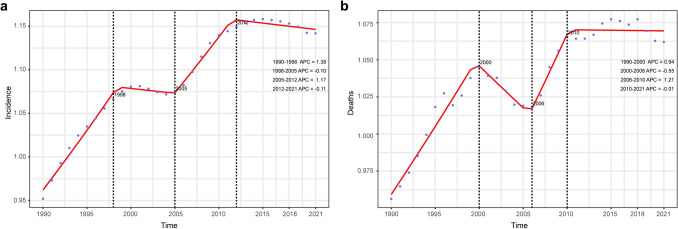
Joinpoint regression analysis of temporal trends of alcoholic liver cancer from 1990 to 2021. The annual percentage change (APC) of the age-standardized morbidity rate (**a**) and mortality rate (**b**)

For age-standardized incidence rate, four time periods were identified with the most noticeable increase observed between 1990 and 1998 (APC = 1.38, *p* < 0.05). The next growth peak occurred in the period 2005–2012, with an APC value of 1.17 (95% CI, 0.98,1.36) (Fig. 5a). The temporal trends of age-standardized mortality rate exhibited similar trends, with three breakpoints in 2000, 2006, and 2010. A significant increase was observed between 1990 and 2000 (APC = 0.94, *p* < 0.05), followed by a substantial decrease between 2000 and 2006 (APC = –0.55, *p* < 0.05). Then, a sharp increase occurred between 2006 and 2010 (APC = 1.21, *p* < 0.05) following a slowly decreasing trend in 2010–2021 (APC = –0.01) (Fig. 5b).

### Age–period–cohort effects

**Fig. 6:**
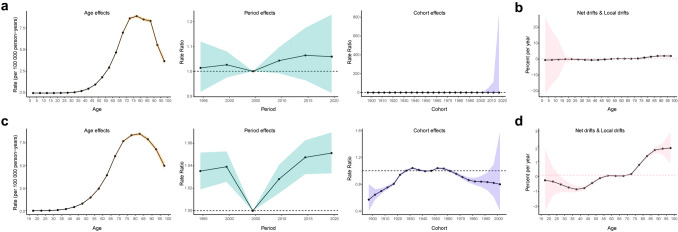
Estimates of age, period, and cohort effects on alcoholic liver cancer. The effects on incidence (**a**), and the local drift and net drift values for different age groups (**b**). The effects on mortality (**c**), and the local drift and net drift values for different age groups (**d**)

Longitudinal age curves revealed that the age-specific rates of incidence increased with age, peaking at 75–79 year age group (8.86), and then gradually decreased. The period effect demonstrated an increasing trend in the period risks of incidence from 1992 to 2021. Compared to the reference period 2002–2006, the relative rate of incidence ranged from 1.01 (0.91–1.12) in the 1992–1997 period to 1.06 (0.96–1.17) in the 2012–2016 period. The global cohort effects exhibited a relatively stable risk trend, the rate ratio ranged from 0.49 in the 1897 cohort to 0.99 in the 1952 cohort, which showed no significant changes (Fig. 6a). The net drift and local drift were then estimated by the age–period–cohort model. The net drift showed an increasing trend in global incidence with a value of 0.21 (95% CI, −0.76 to 1.20). The local drift revealed a shift in the 90–94 year age group (2.05), an increasing trend in the age group below 90–94 years, and a decreasing trend in the age group exceeding 94 years (Fig. 6b).

Age effects on mortality were similar, and the global age-standardized rate increased with age with a delayed peak than the incidence rate, occurring in the 80–84 year age group. The period effect also presented an increased rate ratio compared to the period 2002–2006, and reached a peak of 1.05 (1.03–1.07) in the period 2017–2021. Compared with the birth cohort 1947, the whole cohort effects exhibited a declining risk trend. The cohort effects showed a first trend of increasing and then decreasing, and peaked in the 1950–1954 (1.04) birth cohort (Fig. 6c). The net drift demonstrated a mild increasing trend for from 1990 to 2021 with a value of 0.07 (95% CI, -0.03–0.17). For local drift, a reduction trend occurred from the 15–19 years age group to the 35–39 year age group (-0.86), and then, the mortality increased progressively with age and exhibited a more rapid increase after the 75–79 year age group (Fig. 6d).

## Discussion

The present study provided a comprehensive evaluation of the epidemiological trends of ALC. Our findings demonstrate that the global burden of morbidity and mortality exhibited an increasing trend from 1990 to 2021. Since treatments for viral hepatitis are being devised and continuously improved, alcohol-related liver disease is now becoming a major contributor to liver disease [17]. In this study, ALC accounted for nearly one-fifth of global liver cancer incidence (18.8%) and mortality (18.9%) in 2021, with a significant increase compared to 1990. In contrast to the declining trend in virus-related liver cancer, the result suggests a growing role for alcohol in the epidemiology of liver cancer.

The rising burden can be further explained by the increasing prevalence of alcohol consumption. During the COVID-19, alcohol consumption increased in many areas as a result of isolation and closures [18], and the USA reported a 14% rise in the frequency of alcohol consumption [19], which was expected to increase ALC burden in future. In 2021, alcohol consumption ranked as the ninth leading cause of the global disease burden [20]. Harmful alcohol use is associated with several health problems, including alcohol-associated liver disease (ALD), psychiatric disorders, cardiovascular disease, and neoplasms, among others. As reported, over 200 diseases and injuries are linked to alcohol consumption [21]. It was also reported that the number of people consuming harmful amounts of alcohol increased from 983 million in 1990 to 1.34 billion in 2020, driven by population growth [7]. The global adult per-capita consumption increased from 5.9 L to 6.5 L from 1990 to 2017, and is forecasted to reach 7.6 L (6·5–10·2) by 2030 [22]. Policies that reduce alcohol consumption are essential in mitigating the future risks.

The pronounced geographic heterogeneity in the trend of ALC burden was also verified in the present study. The increased EAPC in Australasia and Southern Latin America reflects complex interactions between rising alcohol consumption, lifestyle changes, and aging populations [23, 24]. Conversely, declines in High-income Asia Pacific may stem from stringent alcohol control policies, effective hepatitis B vaccination programs, and early detection initiatives [25]. The opposite burden trends in high-SDI and low-SDI regions also imply how alcohol consumption interacts with economic development as well as public health capacity. Despite patients in high-SDI regions are more likely to get better education, and to have easier access to healthcare [26], the sustained high level of per-capita alcohol intake caused by professional and stress-related consumption offsetting the protective benefits. Another major driver in high-SDI regions was the elevated synergistic effect of alcohol with other growing epidemic risk factors, such as obesity, smoking, genotoxins exposure, and population aging [27].

Analyzing the gender and age characteristics of morbidity and mortality was an effective way to screen high-risk populations. The gender disparity was identified in this study with males exhibited higher morbidity and mortality rates. It was also reported that three-quarters of alcohol-attributable cancer cases were in males, while the age-standardized incidence rate was three times higher in liver cancer than that in females [28]. This is also in agreement with the previous studies [29, 30]. Gender differences existed in alcohol consumption [31, 32], and data showed that 76.9% of people who consume harmful amounts of alcohol are male [7]. In contrast to female, male drink significantly more alcohol than female, and interestingly, female are more sensitive to alcohol‐related damage than male [33]. Differences in the burden of ALC also existed between different age groups. The age-standardized incidence rate peaked in the 85–89-year-old group for both males and females. The highest burden of age-standardized mortality rate was in the 85–89-year-old group for males, while an early peak in females in the 80–84-year-old group. Considering the effects of aging on primary liver cancer [34], specific medical preventative and treatment methods targeted at this population should be implemented.

The age–period–cohort model was used to clarify the intricate interaction among age, period, and cohort factors. As analyzed, the increased diseased burden of ALC was primarily attributable to age effects. It was also observed that the age effect increased with age with a peak at 75–79 year age group for morbidity, and 80–84 year age group for mortality. This trend may be attributed to the fact that aging stands as a prominent risk factor for cancer [35]. The elderly population exhibits a higher susceptibility to liver cancer compared to younger population [34]. This also aligns with the protracted multistep pathogenesis of hepatitis to cirrhosis to carcinoma as a result of long-term drinking. The period effect suggests that the risk of incidence and mortality increases slightly, suggesting that the prevention and treatment of alcoholic liver cancer still need to be improved. The increased risk was related to the socioeconomic development, lifestyle modifications, and changing alcohol consumption patterns. Alcohol policies over a period of time also affect disease mortality, and the 19 alcohol control policies implemented between 2001 and 2020 reduced all-cause mortality in the Baltic Countries and Poland [36]. The HBV vaccination campaigns launched in China since 1992 also indicated that the period effect had a hysteretic effect on the disease burden of liver cancer [37]. The reduced proportion of HBV-related liver cancer would also affect the proportion of ALC and other etiologies in the future. Owing to different exposure risks to varying degrees of social, natural, and environmental factors, generations born in different eras could exert distinct birth cohort effects [38]. The cohort effect for mortality indicates a declining risk trend since the 1950–1954 birth cohort. The consistent decline in ALC risk reflects the progress made in preventing ALC during these decades. After World War II, with the growth of world population and economic development, the more recent birth cohort experienced a richer economic life with an improved lifestyle and changing drinking habits. This trend was also the result of better health care and stronger health infrastructure, people tend to receive more comprehensive diagnoses and better treatment.

To address the rising disease burden of ALC, policies that reduce alcohol consumption and encourage abstinence are essential in mitigating future risks. It has been reported that the strengthening of alcohol-related public health policies could impact long-term mortality rates of alcohol-attributable hepatocellular carcinoma [39]. Effective government policies should be adopted, including taxation, reduction in utilization capacity, and restrictions on promotion, to lessen the per-capita consumption of alcohol [40, 41]. Based on our results, different interventions should be implemented owing to the regional heterogeneity, high-SDI regions require stronger alcohol control policies. Considering the differences in different age groups and gender, health policies should devote more medical resources to strengthen the welfare of male and the elderly population. Patients with heavy alcohol consumption should be screened as early as possible, especially at 75–79 years. For target regions and populations, addressing these challenges requires a coordinated public health response grounded in health system strengthening, policy innovation, and equitable access to care [42].

There were several inevitable limitations in this current study. First, the analysis was based on the data of ALC in GBD study, the accuracy of the conclusion would be unavoidably confounded by the quality of the used data. Although the GBD study 2021 sought to incorporate as much available data from around the world as possible into its estimates, the quality control of the original data was inevitably affected during collection process, especially in some low-SDI regions and low-income countries, where national systematic surveillance was lacking or insufficient. The scarcity of data in these areas does not reflect well the true picture of their disease burden. As a result, the disease burden of ALC was more likely to be underestimated. Second, regional variations in cancer registry completeness may introduce bias into the estimates [43]. As data were absent or inaccuracy in some regions and countries, the ALC burden was estimated using prediction models or borrowed from nearby countries. Despite the use of robust statistical methods, the estimated data may introduce some degree of uncertainty and affect the accuracy of the result. Thus, we need to interpret the results with caution owing to the data quality and modeling stability. Third, the occurrence and progression of ALC are multifactorial in some cases. Limited by the available data from the GBD database, we could not further evaluate the interplay between alcohol use and other risk factors, such as hepatitis B infections. Finally, the GBD study 2021 lacks detailed insights into specific types and patterns of alcohol consumption. This limitation may limit the depth of understanding of alcoholic liver cancer.

## Conclusion

The overall disease burden of ALC has been increasing over the period 1990–2021. The decomposition analysis revealed that population growth was the primary driver factor. Meanwhile, the disease burden varies across different regions, SDI areas, as well as gender and age groups, especially affecting male and the elderly population. The period effect showed an increasing risk, while improving cohort risks for mortality were identified. The disproportionate burden on males and older adults coupled with regional disparities highlights the urgent need for targeted interventions and healthcare strategies aimed at addressing the growing disease burden.

## Supplementary Information

Below is the link to the electronic supplementary material.

Supplementary file1 (DOCX 23 KB)

## Data Availability

The burden data were downloaded from the GBD study 2021 using the web-based Global Health Data Exchange query tool (http://ghdx.healthdata.org/gbd-results-tool).

## References

[1] Chen L, Zhang J, Peng JY, Yuan Y, Ding Y, Wang Y, et al. Global and country-level analysis of liver cancer: disease burden and recent trends. Curr Med Sci. 2025;45(3):606–615 40512356 10.1007/s11596-025-00064-w

[2] Jiang Z, Zeng G, Dai H, Bian Y, Wang L, Cao W, et al. Global, regional and national burden of liver cancer 1990–2021: a systematic analysis of the global burden of disease study 2021. BMC Public Health. 2025;25(1):931 40057711 10.1186/s12889-025-22026-6 PMC11890516

[3] Tan EY, Danpanichkul P, Yong JN, Yu Z, Tan DJH, Lim WH, et al. Liver cancer in 2021: global burden of disease study. J Hepatol. 2025;82(5):851–860 39481652 10.1016/j.jhep.2024.10.031

[4] McGlynn KA, Petrick JL, El-Serag HB. Epidemiology of hepatocellular carcinoma. Hepatology. 2021;73:4–13 32319693 10.1002/hep.31288 PMC7577946

[5] Aberg F, Jiang ZG, Cortez-Pinto H, Mannisto V. Alcohol-associated liver disease-Global epidemiology. Hepatology. 2024;80(6):1307–1322 38640041 10.1097/HEP.0000000000000899

[6] Radisauskas R, Stelemekas M, Petkeviciene J, Trisauske J, Telksnys T, Miscikiene L, et al. Alcohol-attributable mortality and alcohol control policy in the Baltic Countries and Poland in 2001–2020: an interrupted time-series analysis. Subst Abuse Treat Prev Policy. 2023;18(1):65 37946282 10.1186/s13011-023-00574-7 PMC10636906

[7] Collaborators GBDA. Population-level risks of alcohol consumption by amount, geography, age, sex, and year: a systematic analysis for the Global Burden of Disease Study 2020. Lancet. 2022;400(10347):185–235 35843246 10.1016/S0140-6736(22)00847-9 PMC9289789

[8] Julien J, Ayer T, Tapper EB, Chhatwal J. The rising costs of alcohol-associated liver disease in the United States. Am J Gastroenterol. 2024;119(2):270–277 37463414 10.14309/ajg.0000000000002405 PMC10872874

[9] Zeng RW, Ong CEY, Ong EYH, Chung CH, Lim WH, Xiao J, et al. Global prevalence, clinical characteristics, surveillance, treatment allocation, and outcomes of alcohol-associated hepatocellular carcinoma. Clin Gastroenterol Hepatol. 2024;22(12):2394–2402 38987014 10.1016/j.cgh.2024.06.026

[10] Wang Q, Jia W, Liu J, Zhao Q, Yang Z. Global, regional, and national burden of liver cancer due to alcohol use, 1990–2021: results from the Global Burden of Disease study 2021. Eur J Gastroenterol Hepatol. 2025;37(4):466–476 39621868 10.1097/MEG.0000000000002899

[11] Danpanichkul P, Duangsonk K, Tham EKJ, Tothanarungroj P, Auttapracha T, Prasitsumrit V, et al. Increased mortality from alcohol use disorder, alcohol-associated liver disease, and liver cancer from alcohol among older adults in the United States: 2000 to 2021. Alcohol Clin Exp Res. 2025;49(2):368–378 10.1111/acer.15516 PMC11828968 39701596

[12] Das Gupta P. Standardization and decomposition of rates from cross-classified data. Genus. 1994;50(3–4):171–196 12319256

[13] Cheng X, Tan L, Gao Y, Yang Y, Schwebel DC, Hu G. A new method to attribute differences in total deaths between groups to population size, age structure and age-specific mortality rate. PLoS ONE. 2019;14(5): e0216613 31075117 10.1371/journal.pone.0216613 PMC6510436

[14] Zhang J, Pan L, Guo Q, Lai Y, Liu T, Wang H, et al. The impact of global, regional, and national population ageing on disability-adjusted life years and deaths associated with diabetes during 1990-2019: a global decomposition analysis. Diabetes Metab Syndr. 2023;17(6): 102791 37271078 10.1016/j.dsx.2023.102791

[15] Clegg LX, Hankey BF, Tiwari R, Feuer EJ, Edwards BK. Estimating average annual per cent change in trend analysis. Stat Med. 2009;28(29):3670–3682 19856324 10.1002/sim.3733 PMC2843083

[16] Rosenberg PS, Check DP, Anderson WF. A web tool for age-period-cohort analysis of cancer incidence and mortality rates. Cancer Epidemiol Biomarkers Prev. 2014;23(11):2296–2302 25146089 10.1158/1055-9965.EPI-14-0300 PMC4221491

[17] Fuster D, Samet JH. Alcohol use in patients with chronic liver disease. N Engl J Med. 2018;379(13):1251–1261 30257164 10.1056/NEJMra1715733

[18] Roberts A, Rogers J, Mason R, Siriwardena AN, Hogue T, Whitley GA, et al. Alcohol and other substance use during the COVID-19 pandemic: a systematic review. Drug Alcohol Depend. 2021;229(Pt A): 109150 34749198 10.1016/j.drugalcdep.2021.109150 PMC8559994

[19] Pollard MS, Tucker JS, Green HD Jr. Changes in adult alcohol use and consequences during the COVID-19 pandemic in the US. JAMA Netw Open. 2020;3(9): e2022942 32990735 10.1001/jamanetworkopen.2020.22942 PMC7525354

[20] Collaborators GBDRF. Global burden and strength of evidence for 88 risk factors in 204 countries and 811 subnational locations 1990–2021: a systematic analysis for the Global Burden of Disease Study 2021. Lancet. 2024;403(10440):2162–2203 38762324 10.1016/S0140-6736(24)00933-4 PMC11120204

[21] Belay GM, Lam KKW, Liu Q, Wu CST, Mak YW, Ho KY. Magnitude and determinants of alcohol use disorder among adult population in East Asian countries: a systematic review and meta-analysis. Front Public Health. 2023;11:1144012 36926176 10.3389/fpubh.2023.1144012 PMC10011711

[22] Manthey J, Shield KD, Rylett M, Hasan OSM, Probst C, Rehm J. Global alcohol exposure between 1990 and 2017 and forecasts until 2030: a modelling study. Lancet. 2019;393(10190):2493–2502 31076174 10.1016/S0140-6736(18)32744-2

[23] Medina-Mora ME, Monteiro M, Rafful C, Samano I. Comprehensive analysis of alcohol policies in the Latin America and the Caribbean. Drug Alcohol Rev. 2021;40(3):385–401 33491240 10.1111/dar.13227

[24] Sarich P, Canfell K, Egger S, Banks E, Joshy G, Grogan P, et al. Alcohol consumption, drinking patterns and cancer incidence in an Australian cohort of 226,162 participants aged 45 years and over. Br J Cancer. 2021;124(2):513–523 33041337 10.1038/s41416-020-01101-2 PMC7853127

[25] Zhuang Q, Jin X, Chang Y, Huang H, Tao Z, Lu Z, et al. Burden, trends, and projections of alcohol-associated liver disease and alcohol use disorder in the Asia-Pacific Region 1990-2040. Liver Int. 2025;45(8): e70221 40631455 10.1111/liv.70221

[26] Ranjbari M, Shams Esfandabadi Z, Shevchenko T, Chassagnon-Haned N, Peng W, Tabatabaei M, et al. Mapping healthcare waste management research: past evolution, current challenges, and future perspectives towards a circular economy transition. J Hazard Mater. 2022;15(422): 126724 10.1016/j.jhazmat.2021.126724 34399217

[27] Cano L, Foucher F, Musso O. Geographic diversity of human liver cancers mirrors global social inequalities. Front Oncol. 2025;15:1565692 40452835 10.3389/fonc.2025.1565692 PMC12122337

[28] Rumgay H, Shield K, Charvat H, Ferrari P, Sornpaisarn B, Obot I, et al. Global burden of cancer in 2020 attributable to alcohol consumption: a population-based study. Lancet Oncol. 2021;22(8):1071–1080 34270924 10.1016/S1470-2045(21)00279-5 PMC8324483

[29] Kezer CA, Simonetto DA, Shah VH. Sex differences in alcohol consumption and alcohol-associated liver disease. Mayo Clin Proc. 2021;96(4):1006–1016 33714602 10.1016/j.mayocp.2020.08.020

[30] Choi S, Kim BK, Yon DK, Lee SW, Lee HG, Chang HH, et al. Global burden of primary liver cancer and its association with underlying aetiologies, sociodemographic status, and sex differences from 1990-2019: a DALY-based analysis of the Global Burden of Disease 2019 study. Clin Mol Hepatol. 2023;29(2):433–452 36597018 10.3350/cmh.2022.0316 PMC10121317

[31] Matsushita H, Takaki A. Alcohol and hepatocellular carcinoma. BMJ Open Gastroenterol. 2019;6(1): e000260 31139422 10.1136/bmjgast-2018-000260 PMC6505979

[32] Verplaetse TL, Carretta RF, Struble CA, Pittman B, Roberts W, Zakiniaeiz Y, et al. Gender differences in alcohol use disorder trends from 2009–2019: an intersectional analysis. Alcohol. 2025;123:101–107 39579801 10.1016/j.alcohol.2024.11.003 PMC11871986

[33] Agabio R, Pisanu C, Gessa GL, Franconi F. Sex differences in alcohol use disorder. Curr Med Chem. 2017;24(24):2661–2670 27915987 10.2174/0929867323666161202092908

[34] Macias RIR, Monte MJ, Serrano MA, Gonzalez-Santiago JM, Martin-Arribas I, Simao AL, et al. Impact of aging on primary liver cancer: epidemiology, pathogenesis and therapeutics. Aging (Albany NY). 2021;13(19):23416–23434 34633987 10.18632/aging.203620 PMC8544321

[35] Aunan JR, Cho WC, Soreide K. The biology of aging and cancer: a brief overview of shared and divergent molecular hallmarks. Aging Dis. 2017;8(5):628–642 28966806 10.14336/AD.2017.0103 PMC5614326

[36] Vaitkeviciute J, Gobina I, Janik-Koncewicz K, Lange S, Miscikiene L, Petkeviciene J, et al. Alcohol control policies reduce all-cause mortality in Baltic Countries and Poland between 2001 and 2020. Sci Rep. 2023;13(1):6326 37072446 10.1038/s41598-023-32926-5 PMC10112307

[37] Zheng R, Qu C, Zhang S, Zeng H, Sun K, Gu X, et al. Liver cancer incidence and mortality in China: temporal trends and projections to 2030. Chin J Cancer Res. 2018;30(6):571–579 30700925 10.21147/j.issn.1000-9604.2018.06.01 PMC6328503

[38] Liu C, Zhu S, Zhang J, Wu P, Wang X, Du S, et al. Global, regional, and national burden of liver cancer due to non-alcoholic steatohepatitis, 1990-2019: a decomposition and age–period–cohort analysis. J Gastroenterol. 2023;58(12):1222–1236 37665532 10.1007/s00535-023-02040-4

[39] Diaz LA, Fuentes-Lopez E, Idalsoaga F, Ayares G, Corsi O, Arnold J, et al. Association between public health policies on alcohol and worldwide cancer, liver disease and cardiovascular disease outcomes. J Hepatol. 2024;80(3):409–418 37992972 10.1016/j.jhep.2023.11.006

[40] Morojele NK, Dumbili EW, Obot IS, Parry CDH. Alcohol consumption, harms and policy developments in sub-Saharan Africa: the case for stronger national and regional responses. Drug Alcohol Rev. 2021;40(3):402–419 33629786 10.1111/dar.13247

[41] Stockwell T, Giesbrecht N, Vallance K, Wettlaufer A. Government options to reduce the impact of alcohol on human health: obstacles to effective policy implementation. Nutrients. 2021 Aug 19;13(8). 10.3390/nu13082846 PMC8399748 34445006

[42] Lee MH. Public health strategies for hepatocellular carcinoma: from risk factors to prevention and control. J Liver Cancer. 2025 Jul 28. 10.17998/jlc.2025.07.25 PMC12518992 40721214

[43] Diseases GBD, Injuries C. Global incidence, prevalence, years lived with disability (YLDs), disability-adjusted life-years (DALYs), and healthy life expectancy (HALE) for 371 diseases and injuries in 204 countries and territories and 811 subnational locations, 1990–2021: a systematic analysis for the Global Burden of Disease Study 2021. Lancet. 2024;403(10440):2133–2161 38642570 10.1016/S0140-6736(24)00757-8 PMC11122111

