# Supplementary material for: Epidemiological trends and age–period–cohort analysis of alcoholic liver cancer from 1990 to 2021
Source: Hepatol Int. 2025 Dec 20;20(2):330–41. doi: 10.1007/s12072-025-10966-5 (PMC13121189; doi:10.1007/s12072-025-10966-5)
Supplement: Supplementary file 1 — Supplementary file1 (DOCX 23 KB) [file 12072_2025_10966_MOESM1_ESM.docx]

**Table S1** The global burden of alcoholic liver cancer in 1990 and 2021, and changes in data of incidence, prevalence, deaths and, DALYs.

|  |  | Total | | | Male | | | Female | | |
| --- | --- | --- | --- | --- | --- | --- | --- | --- | --- | --- |
|  |  | 1990 | 2021 | change | 1990 | 2021 | change | 1990 | 2021 | change |
| Incidence | Number | 38445.23  (31540.01,  6398.95) | 99543.67  (80957.40,  120401.87) | 158.92  (135.19,  188.10) | 29520.26  (24356.04,  35926.08) | 77933.33  (64115.95,  94162.85) | 164.00  (137.03,  200.83) | 8924.98  (7052.40,  11155.13) | 21610.34  (17127.62,  26522.38) | 142.13  (109.78,  180.50) |
|  | ASR | 0.95  (0.78,1.14) | 1.91  (1.57,2.29) | 0.55  (0.47,0.63) | 1.58  (1.32,1.91) | 1.14  (0.93,1.38) | 0.55  (0.47,0.63) | 0.42  (0.33,0.52) | 0.47  (0.37,0.57) | 0.41  (0.35,0.47) |
| Prevalence | Number | 44847.95  (37054.85,  4443.66) | 132033.13  (107264.09,  159049.29) | 194.40  (167.43,  226.06) | 34958.75  (29286.08,  42514.28) | 105628.78  (87084.21,126662.40) | 202.15  (172.20,  241.83) | 9889.20  (7870.73,  12250.23) | 26404.35  (21106.55,  32298.16) | 167.00  (130.99,  207.93) |
|  | ASR | 1.09  (0.90,1.31) | 1.51  (1.23,1.82) | 1.10  (0.96,1.24) | 1.80  (1.51,2.17) | 2.56  (2.10,3.06) | 1.16  (1.01,1.32) | 0.46  (0.36,0.57) | 0.57  (0.46,0.70) | 0.79  (0.70,0.88) |
| Deaths | Number | 38171.63  (31169.88,  46199.72) | 92227.78  (75053.11,  112160.27) | 141.61  (118.82,  169.97) | 29020.39  (23917.61,  35445.06) | 71267.67  (58194.93,  86612.55) | 145.58  (120.16,  181.45) | 9151.24  (7227.65,  11508.44) | 20960.11  (16643.10,  25767.86) | 129.04  (98.63,  165.84) |
|  | ASR | 0.95  (0.78,1.15) | 1.06  (0.86,1.29) | 0.31  (0.25,0.37) | 1.59  (1.32,1.93) | 1.77  (1.45,2.13) | 0.29  (0.22,0.35) | 0.43  (0.34,0.54) | 0.45  (0.36,0.56) | 0.23  (0.18,0.29) |
| DALYs | Number | 1042115.90  (852871.47,  1280543.70) | 2316027.04  (1887012.95,  2845788.99) | 122.24  (99.82,  150.43) | 805085.63  (661941.86,  991046.01) | 1816366.02  (1477467.38,  2246605.99) | 125.61  (101.15,  161.44) | 237030.26  (187914.90,295374.59) | 499661.02  (398674.46,613742.62) | 110.80  (80.75,  145.63) |
|  | ASR | 25.03  (20.59,30.50) | 26.39  (21.53,32.28) | 0.12  (0.06,0.19) | 40.82  (33.87,49.76) | 43.36  (35.34,53.29) | 0.13  (0.06,0.20) | 10.89  (8.62,13.62) | 10.90  (8.73,13.38) | 0.03  (-0.02,0.08) |

ASR: age standardized rate, DALYs: disability adjusted life years. Percentage change was used to evaluate changes in number, and the estimated annual percentage change was used to assess changes in ASR.
